# Network pharmacology and experiments verify the effect of triptolide on extraocular muscle fibrosis

**DOI:** 10.1371/journal.pone.0336487

**Published:** 2025-11-10

**Authors:** Minmin Jiang, Ping Wang, Dandan Yu, Panpan Guo, Zhiyu Qu, Jingxiao Zhao, Shuxun Yan

**Affiliations:** Department of Endocrinology, The First Affiliated Hospital of Henan University of Chinese Medicine, Zhengzhou, Henan Province, China; Jan Biziel University Hospital No 2 in Bydgoszcz: Szpital Uniwersytecki Nr 2 im dr Jana Biziela w Bydgoszczy, POLAND

## Abstract

Drugs usually do not prevent extraocular muscle fibrosis in Graves’ ophthalmopathy (GO), and surgical treatment has complications and does not cure extraocular muscle fibrosis. Triptolide (TPL) has shown antifibrotic effects; however, the mechanism by which it treats extraocular muscle fibrosis in GO remains unclear. The aim of this study was to investigate the therapeutic effect and potential mechanism of TPL through a combination of network pharmacology and experimental validation. Network pharmacology identified 10 potential therapeutic targets, 1767 gene ontology terms, and 95 signaling pathways, including the PI3K/AKT pathway. Molecular docking revealed a strong affinity between core targets on the PI3K/AKT pathway and TPL. The experimental results showed that TPL inhibited the proliferation of OFs in vitro in a concentration-dependent manner. It significantly inhibited the expression of TGF-β1-induced fibrosis-related markers, such as FN, CTGF, α-SMA, and TIMP-1, while significantly down-regulating the expression of PI3K/AKT signaling proteins. The use of inhibitors of the PI3K/AKT pathway inhibited the expression of fibrosis-related markers. These findings suggest that TPL can resist extraocular muscle fibrosis in GO through multiple pathways, in which the PI3K/AKT pathway plays a key role.

## Introduction

Extraocular muscle fibrosis causes muscle tissue to lose its normal contractile ability, leading to restricted eye movement, and patients may experience strabismus, diplopia, or even optic nerve compression leading to blindness [1,2]. Extraocular muscle fibrosis is a key pathological feature of Graves’ ophthalmopathy (GO), the most common clinical orbital disease [3]. Existing treatments are mostly focused on the active phase of GO [4]. For inactive (fibrotic) GO, most surgical treatments are used, and there is a lack of effective therapeutic drugs, but surgery only reduces strabismus, relieves eyelid recession and optic nerve compression [5], and does not cure extraocular muscle fibrosis. Moreover, the success rate of strabismus surgery is variable, with about a quarter of patients requiring reoperation [6]. Eye surgery can cause postoperative complications such as scleral perforation, retinal detachment, recurrent or continuous postoperative diplopia, overcorrection, and under-correction of the eyelids [7,8], which aggravate the psychological and financial burden of patients.

**Fig 1:**
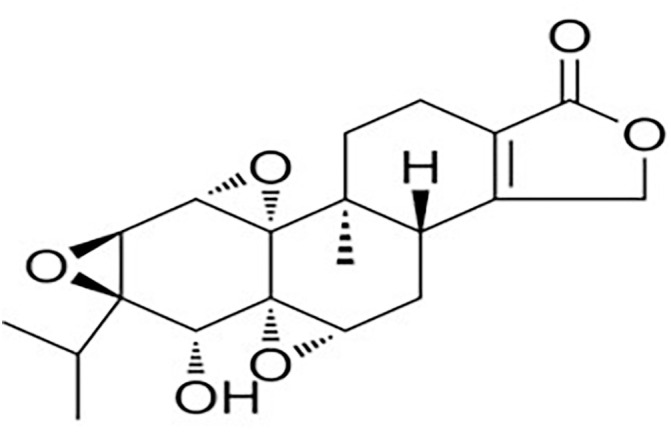
The structure of triptolide.

Triptolide (TPL) (Fig 1) is a diterpenoid tricyclic oxide that is extracted from the root of *Tripterygium wilfordii Hook. F*, a Chinese medicine [9]. It has beneficial effects such as anticancer [10], anti-inflammatory [11], anti-oxidative stress [12], and immune modulation [13]. Several studies have demonstrated the therapeutic effects of TPL on organ fibrosis (e.g., heart, lung, and kidney) [14–16]. Previous studies have shown that TPL can treat active GO by inhibiting IFN-γ-induced overexpression of HLA-DR, CD40, hyaluronic acid, and intercellular adhesion molecule (ICAM)-1 in orbital fibroblasts (OFs) [17]. However, its effects on extraocular muscle fibrosis in GO and the underlying mechanisms have yet to be explored.

Network pharmacology provides new perspectives and methods for Chinese medicine research by revealing and visualizing the potential interaction networks between traditional Chinese medicine and multifactorial diseases [18]. Its integration with molecular docking analysis enhances understanding of the binding affinity and interaction mechanisms between selected compounds and key protein targets, thereby validating research findings [19]. Here, we used network pharmacology and molecular docking to study the effects and specific mechanisms of TPL on extraocular muscle fibrosis in GO, and verified these findings using in vitro models.

## Materials and methods

### TPL target acquisition

Using “*Tripterygium wilfordii Hook. F*” as the keyword, its composition was queried in the TCMSP database (TCMSP, https://tcmspw.com/tcmsp.php). The OB and DL of TPL in the search results were 51.29% and 0.68, respectively. The screening criteria of OB ≥ 30% and DL ≥ 0.18 were met [20]. Then, the target information of TPL was screened from the TCMSP database, and the target names were entered into the Uniprot (http://www.uniprot.org/) database to obtain standardized gene symbols.

### Prediction of GO and extraocular muscle fibrosis-related targets

The disease targets were obtained by using “Graves’ ophthalmopathy” and “extraocular muscle fibrosis” as keywords in OMIM database (OMIM, https://omim.org), PharmGkb database (https://www.pharmgkb.org/), GeneCards database (https://www.genecards.org), and treatment target database (TTD, https://db.idrblab.net/ttd/). By integrating the targets identified in the four databases, we eliminated duplicates and standardized the final targets.

### Protein-protein interaction (PPI) network construction

Intersected TPL targets with disease targets to obtain potential targets of drug intervention in disease. To obtain protein-protein interaction relationships, potential targets were imported to the STRING website (https://string-db.org). Then the results were imported into Cytoscape software, and the network analysis tool was applied to analyze the targets for analysis to analyze and construct PPI network.

### Gene ontology and pathway enrichment analysis

The obtained intersected gene symbols were converted to relevant Entrez IDs, followed by KEGG pathway enrichment analysis and gene ontology enrichment analysis. Gene ontology analysis involves cellular components (CC), biological processes (BP), and molecular functions (MF). Further study of relevant R packages based on R software, such as ggplot2, enrichplot, pathview, Seurat, DOSE, stringi, clusterProfiler, BiocManager, and org.Hs.e.g.,db. Only when the q-value was less than 0.05, the corresponding functional term and pathway were recognized as statistically significant and retained for further analysis.

### Molecular docking validation

First, the 3D structures of TPL were retrieved from the PubChem database and downloaded in SDF format. Subsequently, these structures were optimized in Chem3D 18.0 software. Next, the entries of the key targets were determined using the UniProt database, and the corresponding ligand structures were retrieved from the PDB database based on these entries. Afterwards, the optimized compound and ligand files were imported into AutoDock for preprocessing, including removing water from the target protein and adding polar hydrogen atoms to form the active site, and converting all the files to PDBQT format. Next, the 3D coordinates and grid parameters of the target protein are adjusted to optimize the positioning of the binding site. Finally, ten molecular dockings were performed by AutoDock Vina, and the results with the lowest binding energy were selected after each docking. Lower docking scores indicate a stronger binding affinity of the small molecule to the ligand.

### Primary cell culture

Normal human primary OFs were purchased from icell (Shanghai) Biotechnology Technology Co. Ltd (icell, China). OFs were cultured with DMEM medium containing 10% FBS at 37°C and 5% CO2, in a humidified environment. As in previous studies, we used TGF-β1 to construct an in vitro model [21]. Cells were stimulated with 10 ng/mL TGF-β1 (Peprotech, USA) with or without TPL (Yuanye, China) and 10 μM LY294002 (MCE, USA).

### CCK-8 assay

To evaluate cell viability, the CCK-8 Assay Kit (GLPBIO, USA) was used according to the manufacturer’s instructions. OFs were incubated with different concentrations (0, 1, 5, 10, 20, 50, 100, 200 pM) of TPL for 24 h or 48 h. Cell viability was based on the formula below.


\footnotesizeCell viability (%)=[(experimental hole absorbance−blank hole absorbance)/(control hole absorbance−blank hole absorbance)]×100

### Western blot

To obtain cell lysates, OFs were treated with RIPA lysis solution (servicebio, China) supplemented with protease and phosphatase inhibitors. Determination of protein concentration using the Bicinchoninic Acid Assay (BCA) assay kit (Beyotime, China). Equal amounts of total proteins were separated by electrophoresis on FuturePAGE™ 4–20% precast gels and by electrotransfer to PVDF membranes (Millipore, Germany). To block any non-specific binding, membranes were blocked with 5% skimmed milk powder (DEVONDALE, Australia) for 1.5h, followed by overnight incubation with primary antibodies against p-AKT (CST, USA), AKT (CST, USA), β-actin (Peprotech, China), p-PI3K (affinity, USA), and PI3K (Genetex, USA). β-actin was used as an endogenous control. After washing the membranes with tris-buffered saline-Tween 3 times, they were incubated with horseradish peroxidase-coupled sheep anti-rabbit secondary antibody for 1.5 h. Finally, membranes were incubated with the ECL Ultrasensitive Luminescence Kit (servicebio, China) and imaging was captured using a chemiluminescence imager (Biorad, USA). The acquired images were analyzed using ImageJ software.

### RT-qPCR

Total RNA was extracted with TRIzol reagent (Ambion, USA), after which RNA concentration, quality, and integrity were assessed by NanoDrop spectrophotometer (Mio, China). The RNA was then reverse transcribed to cDNA with the HiScript II Q Select RT SuperMix for qPCR kit (Vazyme, China). RT-PCR was conducted using the 2*Q3 SYBR qPCR Master Mix kit (ToloBio, China). To determine the relative expression levels, β-actin was used as an endogenous control. The 2 ^-ΔΔCT^ method was used for analysis. The primer sequences that were used in this study are listed in Table 1.

**Table 1:** RT-PCR primer sequences.

Gene name	Primer sequence
β-actin	Forward: 5’-CCCTGGAGAAGAGCTACGAG-3’
	Reverse: 5’-CGTACAGGTCTTTGCGGATG-3’
α-SMA	Forward: 5’-TCATGGTCGGTATGGGTCAG-3’
	Reverse: 5’-CGTTGTAGAAGGTGTGGTGC-3’
TIMP-1	Forward: 5’-CCTGTTGTTGCTGTGGCTGATA-3’
	Reverse: 5’-TGGTCTGGTTGACTTCTGGTGT-3’
FN	Forward: 5’-ACGACTCCCTTTTCTCCTCTTG-3’
	Reverse: 5’-GGTACTGTGGCTCATCTCCCTC-3’
CTGF	Forward: 5’-CCGTACTCCCAAAATCTCCA-3’
	Reverse: 5’-GTAATGGCAGGCACAGGTCT-3’

### Statistical analysis

Each experiment was repeated three times in an independent manner, and the data were presented as mean ± standard deviation and were statistically analyzed and graphed using GraphPad Prism. When comparing multiple groups, all the data conform to normal distribution, and the variance between groups is not similar, so one-way ANOVA is used. A p-value of less than 0.05 was regarded as statistically significant.

## Results

### Identification of targets and crossover genes

**Fig 2:**
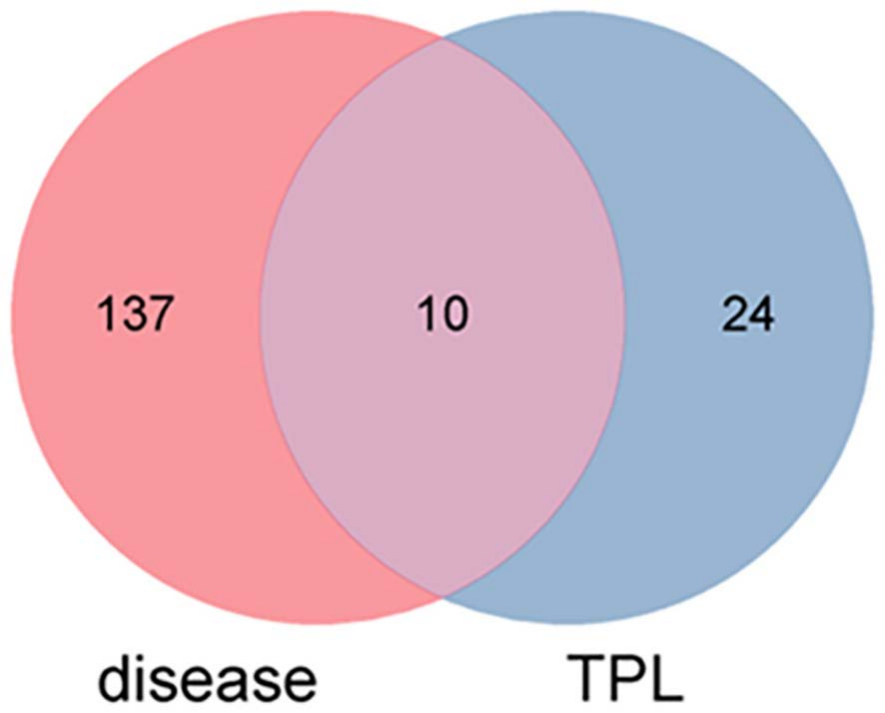
Venn diagram of intersecting targets of TPL and extraocular muscle fibrosis in GO.

Based on a search in the TCMSP database, 34 TPL-related targets were identified. The number of GO and extraocular muscle fibrosis targets from GeneCards, OMIM, TTD, and PharmGkb databases was 370 and 2,428, respectively. GO and extraocular muscle fibrosis intersected with each other, yielding 147 targets. As revealed in the Venn diagram (Fig 2), the number of targets common to TPL, GO, and extraocular muscle fibrosis was 10.

### PPI network graph analysis

**Fig 3:**
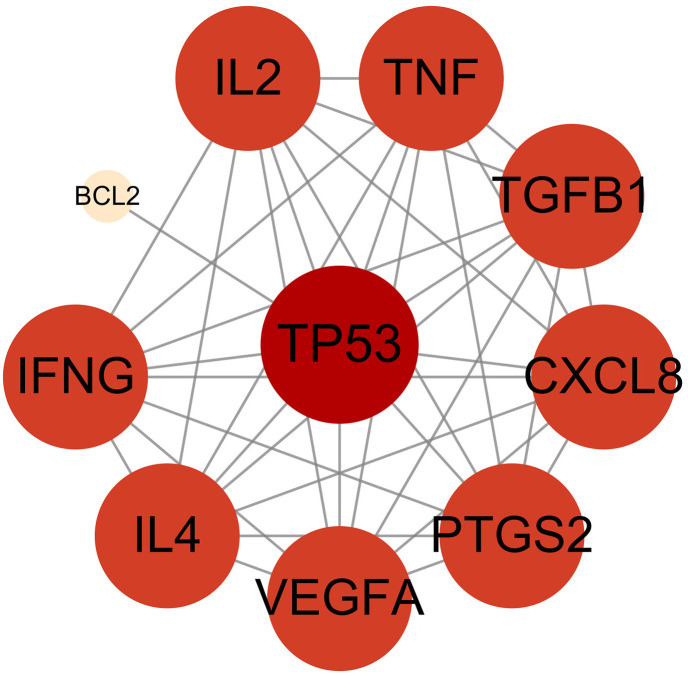
PPI network at the intersection target of TPL-Disease. Note: The nodes in the figure represent target proteins, the edges represent connections between targets, and the orange to red color indicates the degree value from small to large. The higher the importance of a node, the larger and redder the node is.

Using STRING database and Cytoscape software, we successfully constructed a PPI network graph, including 10 nodes and 37 edges. The 10 intersecting targets were analyzed using the networker analysis program, and the results are shown in Fig 3. In addition to BCL2, all targets may be the core targets of TPL for the treatment of extraocular muscle fibrosis in GO.

### Gene ontology and pathway enrichment analysis

**Fig 4:**
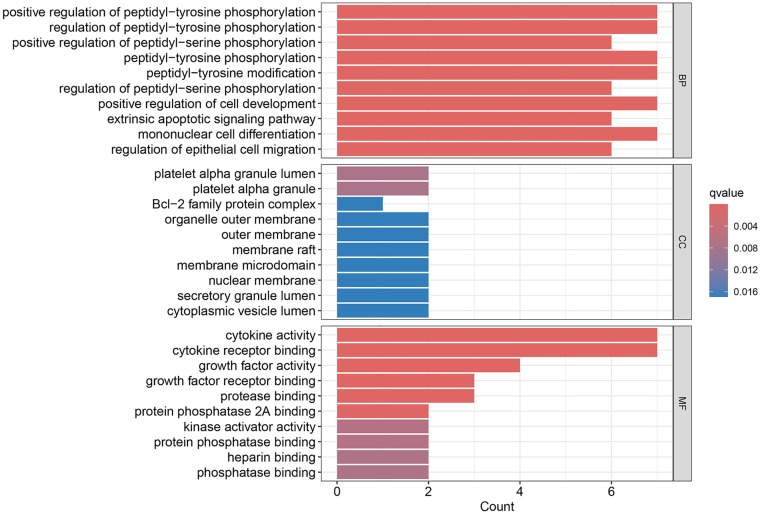
Gene ontology analysis.

Gene ontology and KEGG analysis of the intersected targets was performed using R software. The gene ontology analysis showed that the number of BP items was 1,684, the number of CC items was 23, and the number of MF items was 60. The top 10 significant items of each module are shown in Fig 4. Among BP, TPL was mainly associated with regulation of peptide-tyrosine phosphorylation, positive regulation of peptidyl-serine phosphorylation and positive regulation of peptidyl-tyrosine phosphorylation. Among CC, platelet alpha granules, platelet alpha granule lumen, and Bcl-2 family protein complexes are associated with TPL. Among MF, growth factor activity, cytokine receptor binding and cytokine activity were associated with TPL treatment of extraocular muscle fibrosis in GO.

**Fig 5:**
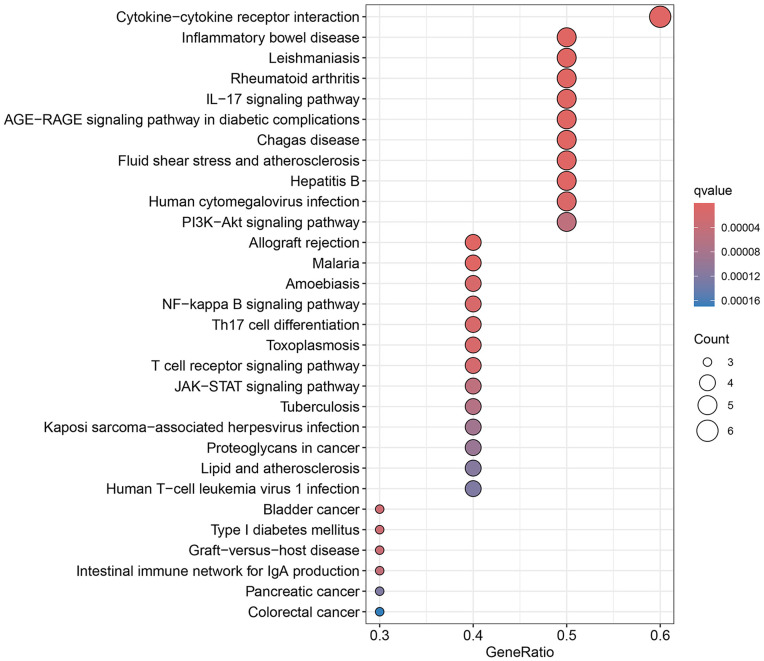
KEGG analysis.

**Fig 6:**
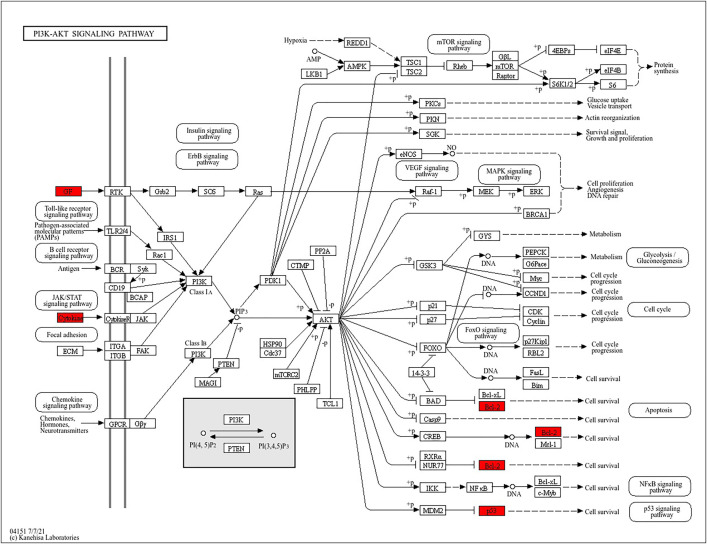
PI3K/AKT signaling pathway. Note: The red in the figure represents the intersection target of TPL-disease. Cytokines include IL-4, IL-2 and TP53, and GF refers to growth factors such as VEGFA.

KEGG pathway enrichment analysis yielded 95 results. The top 30 significant items were selected and shown in Fig 5. TPL may work together through multiple pathways to treat extraocular muscle fibrosis in GO. The major pathways involved PI3K/AKT signaling pathway, Th17 cell differentiation, cytokine-cytokine receptor interaction, and IL-17 signaling pathway, etc. The PI3K/AKT signaling pathway is shown in Fig 6, the TPL- disease targets in PI 3 K/AKT pathway include IL 4, IL 2, TP 53, VEGFA and BCC 2.

### Molecular docking

**Fig 7:**
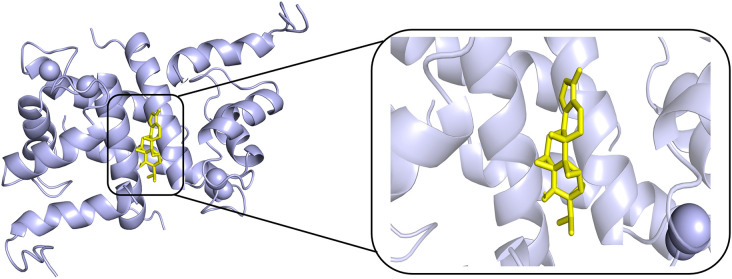
Molecular docking of Triptolide with TP53.

**Fig 8:**
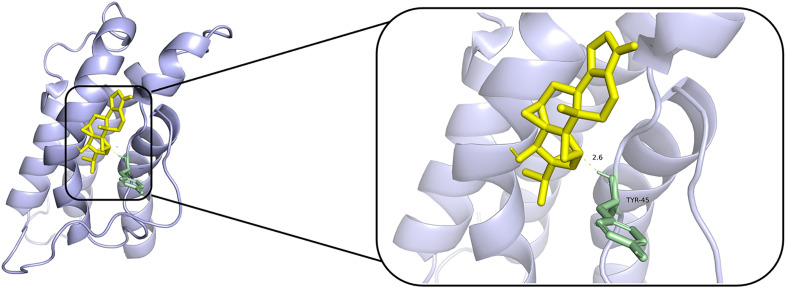
Molecular docking of Triptolide with IL2.

**Fig 9:**
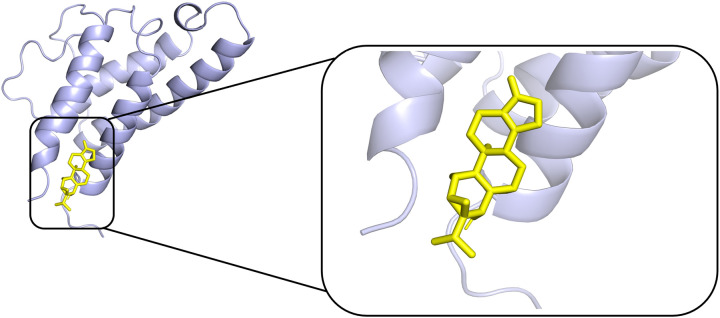
Molecular docking of Triptolide with IL4.

**Fig 10:**
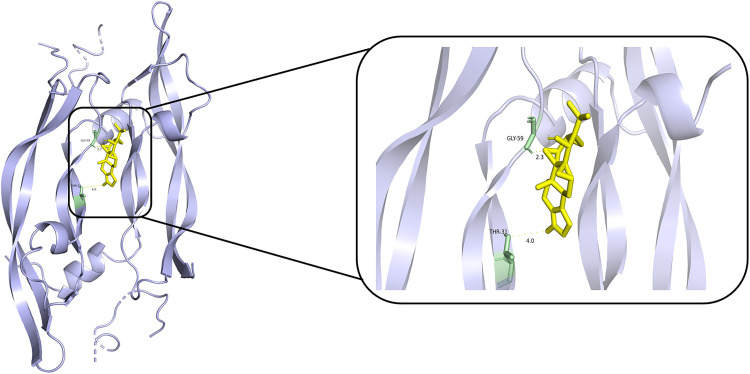
Molecular docking of Triptolide with VEGFA.

According to PPI network and KEGG results, we conducted molecular docking of core proteins (IL4, TP53, IL2, and VEGFA) on the PI3K/AKT pathway with TPL. The docking results are presented in Table 2 and Figs 7, 8, 9, 10. According to Table 2, the binding energies of IL4, TP53, IL2 and VEGFA to TPL were less than −5 kcal/mol. This suggests that the compound has a good affinity with the core target gene [22].

**Table 2:** The results of molecular docking.

Protein	Ligands	Affinity (kcal/mol)
TP53	Triptolide	−7.5
IL2	Triptolide	−6.9
VEGFA	Triptolide	−6.7
IL4	Triptolide	−6.4

### Effect of TPL on cell viability

**Fig 11:**
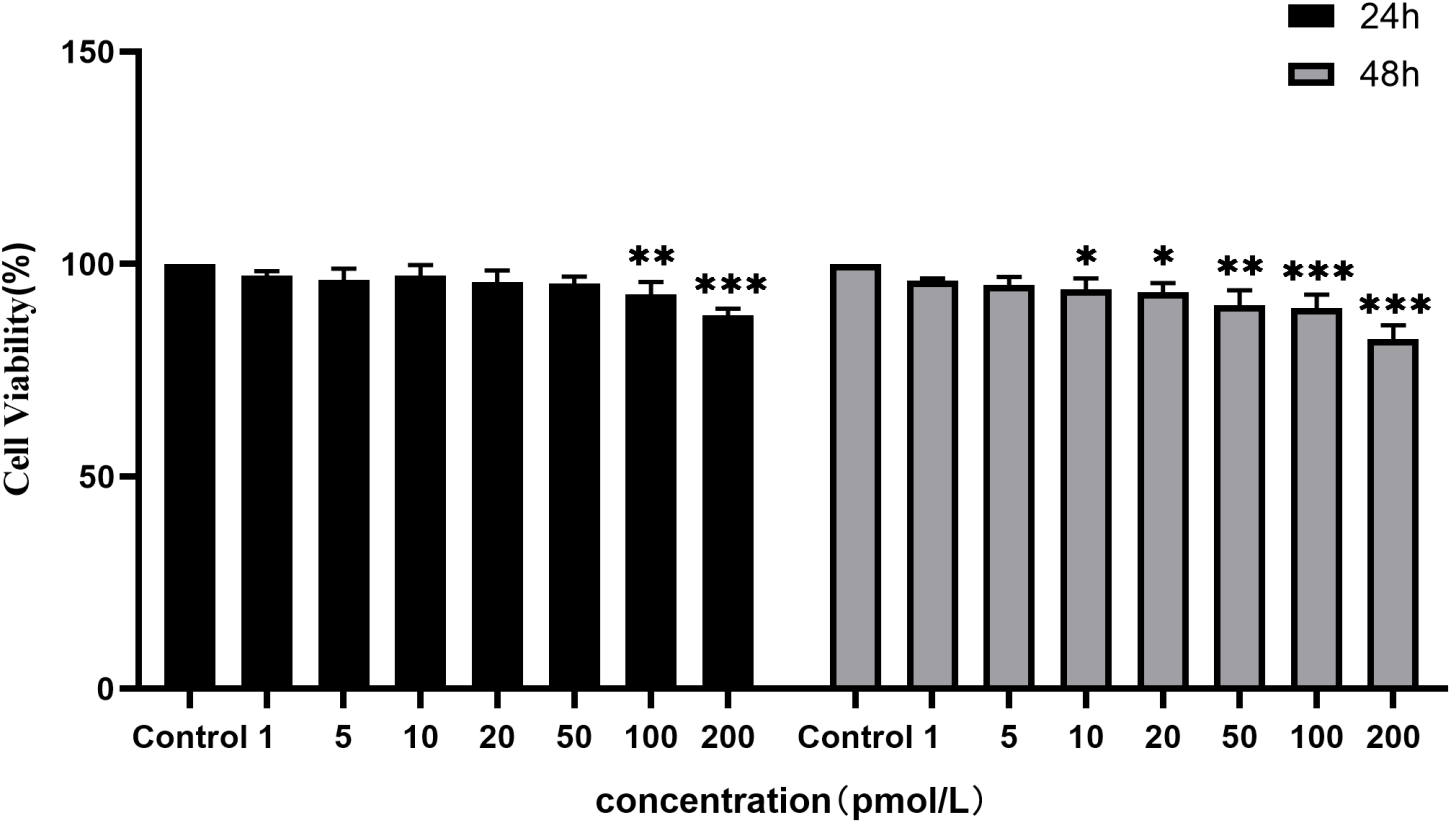
Effect of TPL on the viability of OFs. Note: Compared with the 0 pM group, *P < 0.05, **P < 0.01, ***P < 0.001.

The influence of TPL on cell viability was examined by using the CCK-8 assay. As presented in Fig 11, the inhibitory effect of TPL on the activity of OFs was dose-dependent. Compared with the control group (0 pM TPL), the cell activity was significantly decreased after 24 h of intervention with ≥100 pM TPL (*P* < 0.05). When the intervention time was prolonged to 48 h, 10 pM TPL (*P* < 0.05) had already clearly demonstrated the inhibitory effect on cell activity. Therefore, 5 pM TPL was selected as the intervention concentration for all subsequent experiments.

### Effect of TPL on TGF-β1-induced fibrosis of OFs

**Fig 12:**
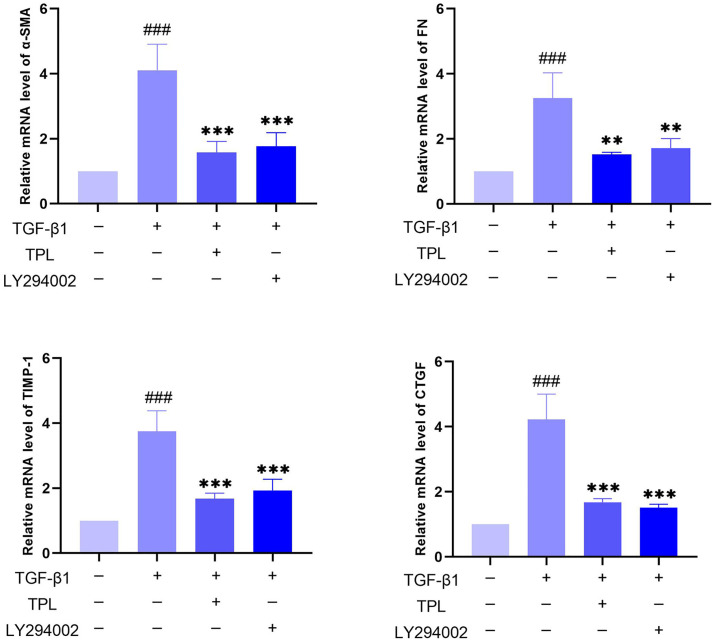
Effect of TPL on fibrotic mRNAs. Note: compared with TGF-β1(-) group, ^###^ P < 0.001; compared with TGF-β1(+) group, **P < 0.01, ***P < 0.001.

RT-PCR was used to assay the effects of TPL and PI3K inhibitors on TGF-β1-induced fibrosis in OFs. As illustrated in Fig 12, TGF-β1 induced fibrotic mRNA expression, such as α-SMA, TIMP-1, FN, and CTGF, compared with the control group (*P* < 0.001); the use of TPL and LY294002 inhibited TGF-β1-stimulated α-SMA, TIMP-1, FN, and CTGF mRNA expression (*P* < 0.01). It is suggested that TPL may inhibit OFs fibrosis by inhibiting PI3K/AKT signal.

### Effect of TPL on PI3K/AKT pathway in TGF-β1-induced OFs

**Fig 13:**
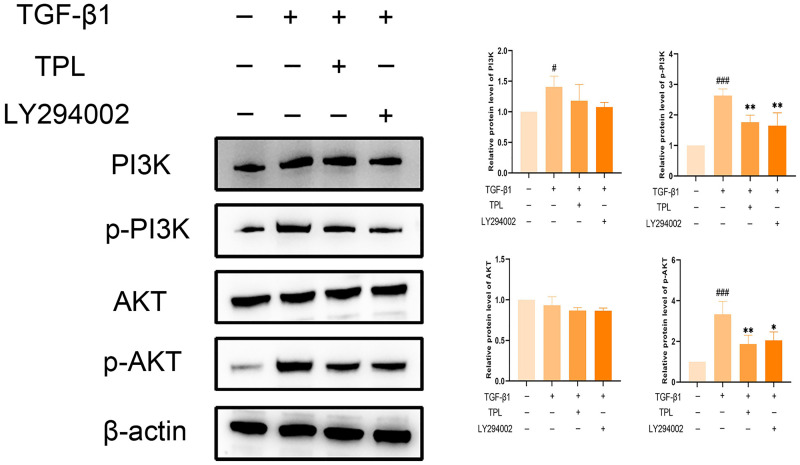
Effect of TPL on PI3K/AKT pathway related proteins. Note: Compared with the TGF-β1(-) group, ^#^ P < 0.05, ^# # #^ P < 0.001; Compared with TGF-β1(+) group, **P < 0.01, ***P < 0.001.

The effect of TPL on the PI3K/AKT pathway in TGF-β1-induced OFs was detected by western blot. As shown in Fig 13, the stimulation of TGF-β1 significantly up-regulated the protein levels of p-PI3K, PI3K, and p-AKT compared with the control group (*P* < 0.001); both TPL and LY294002 significantly decreased the protein expression of p-AKT and p-PI3K induced by TGF-β1 (*P* < 0.01). It suggests that TPL may inhibit extraocular muscle fibrosis in GO by suppressing PI3K/AKT signaling.

## Discussion

Extraocular muscle fibrosis in GO can lead to disfiguring proptosis and dysfunction. This places a great financial burden on patients and society [23]. However, the pathogenesis of GO is unknown; there is a lack of good therapeutic options for treating fibrosis in GO, and medications usually do not prevent the development of advanced extraocular muscle fibrosis [24]. Therefore, it is important to discover new drugs for the management of extraocular muscle fibrosis in GO and investigate the related therapeutic mechanisms.

Based on previous studies, we explored the impact of TPL against extraocular muscle fibrosis in GO. The network pharmacology study obtained 10 potential targets, which were TGFB1, TP53, PTGS2, CXCL8, IL2, IFNG, IL4, VEGFA, TNF, and BCL2. According to the PPI network analysis, TPL could treat extraocular muscle fibrosis in GO through key targets including IL4, TP53, IL2, and VEGFA. Studies have shown that IL-4 is predominant in GO, and it blocks IL-1β-induced expression of tissue inhibitor of metalloproteinases-1 (TIMP-1) involved in fibrosis [25,26]. Some studies have reported that TP53 gene polymorphisms are associated with GD, which has a strong association with GO [27]. TP53 dysregulation has also been associated with the development of fibrotic diseases [28]. In addition, the IL2RA/rs2104286 locus has been associated with susceptibility to GO [29]. Studies have shown that VEGFA is highly expressed in GO patients [30]. The expression of VEGFA receptor VEGFR-2 mRNA in orbital tissues of patients with inactive GO (characterized by muscle bundle atrophy and fibrosis) was increased by 2.53 ± 1.10-fold compared with controls [31,32].

Gene ontology enrichment analysis showed that TPL exerts its therapeutic effects mainly by acting on cytokine activity, cytokine receptor binding and growth factor activity. KEGG pathway analysis indicated that among the signaling pathways associated with extraocular muscle fibrosis in GO, the PI3K/AKT signaling pathway was highly enriched and appeared to be the most critical pathway. In addition, molecular docking validation results showed that core proteins IL4, TP53, IL2, and VEGFA on the PI3K/AKT pathway have good affinity with TPL. The PI3K/AKT signaling pathway is a classical signaling pathway that can play a role in regulating the inflammatory response, oxidative stress, cellular autophagy, and metabolism by modulating multiple downstream targets such as mTOR [33,34]. This pathway is initiated by the combination of extracellular growth factors such as VEGFA or cytokines (including IL-4, IL-2, and TP53) with their corresponding cell surface receptors (such as receptor tyrosine kinase (RTK) or G protein-coupled receptor (GPCR)), which leads to the activation of PI3K [35–37]. This recruits and activates AKT, which phosphorylates downstream targets by translocating to cellular compartments such as the nucleus, mitochondria, and endoplasmic reticulum [37]. Studies have demonstrated that the PI3K/AKT signaling pathway is aberrantly expressed in GO [38,39]. OFs and fibroblasts masquerading as OFs are important players in GO, and OFs can differentiate into myofibroblasts, which mediate extracellular matrix deposition and ultimately lead to tissue fibrosis [40,41]. Thyrotropin receptor (TSHR), an important antigen of GO, can stimulate the proliferation of OFs through PI3K/AKT signaling [42]. Yang et al [43] showed that activation of PI3K signaling mediated the process of macrophages promoting hyaluronic acid secretion from GO-OFs and facilitated the generation of a pro-fibrotic phenotype. Thereafter, Cao et al [44] reported that AKT signaling is involved in TGF-β1 stimulation of OFs to express α-SMA, a typical marker of myofibroblast differentiation, and fibrotic proteins such as FN. In addition, inhibition of PI3K/AKT and downstream mTOR signaling can inhibit OFs fibrosis and treat extraocular muscle fibrosis in GO [24,45,46]. In this study, we used TGF-β1 to stimulate OFs to construct an in vitro fibrosis model. TPL intervention can obviously inhibit the fibrosis markers such as α-SMA, TIMP-1, CTGF and FN produced by TGF-β1 stimulation. In addition, we report for the first time that TGF-β1 can promote PI3K/AKT signal transduction in OFs. TPL treatment can significantly reduce the relative protein expression of p-PI3K and p-AKT. The application of PI3K/AKT pathway inhibitor LY294002 can significantly inhibit the production of fibrosis markers such as α-SMA, TIMP-1, CTGF, and FN. This is consistent with previous findings [24]. This indicates that TPL may play a role in the process of extraocular muscle fibrosis in GO by influencing the PI3K/AKT signaling pathway.

However, the study has some limitations. We only focused on the impact of TPL on extraocular muscle fibrosis in vitro and did not conduct an in vivo study. TPL has a variety of toxicities, such as organ toxicity, cytotoxicity, and immunotoxicity [47,48]. However, many studies also show that low dose TPL has no obvious toxicity, and toxic dose TPL under physiological conditions has protective effect on organs under pathological conditions [49,50]. Therefore, before TPL is used to treat GO, it is essential to study the animal model of GO and the treatment of human beings. The study should also determine the safety, efficacy, and non-toxic dose range of specific target organs and diseases, clarify the ways and mechanisms of exposure change, and establish a toxicity early warning system. In addition, TPL derivatives such as Minnelide have demonstrated a high clinical safety [51], and may be promising clinical agents.

## Conclusion

In this study, we investigated the role and mechanism of TPL in treating extraocular muscle fibrosis in GO through network pharmacological prediction and molecular docking. In addition, related in vitro experiments were carried out to verify these findings. TPL inhibited the proliferation of OFs in a concentration-dependent manner. It inhibits OFs fibrosis through the PI3K/AKT pathway. This study provides theoretical support and experimental evidence for the clinical use of TPL in the treatment of GO, and emphasizes its potential as a therapeutic agent for GO in the fibrosis stage.

## Supporting information

S1 File S1 raw images. (PDF)

S2 File S2 raw data. (ZIP)
