# Supplementary material for: Network pharmacology and experiments verify the effect of triptolide on extraocular muscle fibrosis
Source: PLoS One. 2025 Nov 10;20(11):e0336487. doi: 10.1371/journal.pone.0336487 (PMC12599947; doi:10.1371/journal.pone.0336487)
Supplement: S1 File — (PDF) [file pone.0336487.s001.pdf]

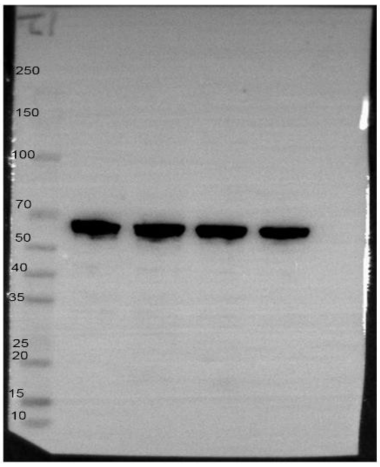

the first AKT

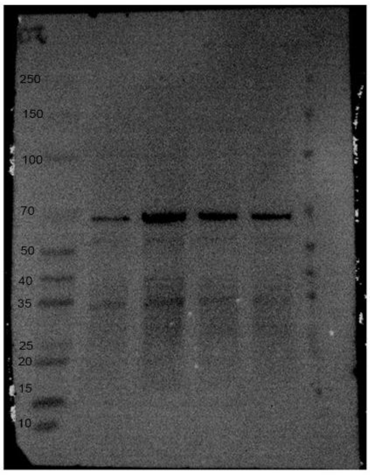

the first p-AKT

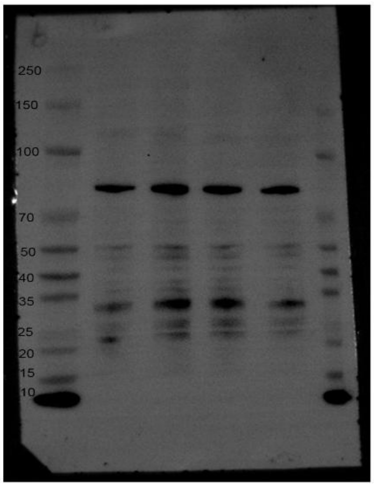

the first PI3K

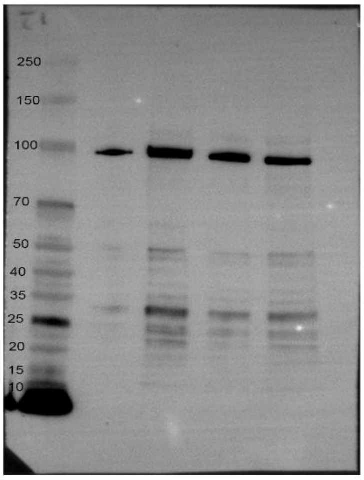

the first p-PI3K

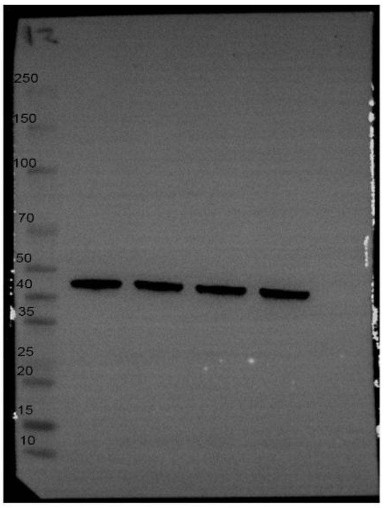

the first β-actin

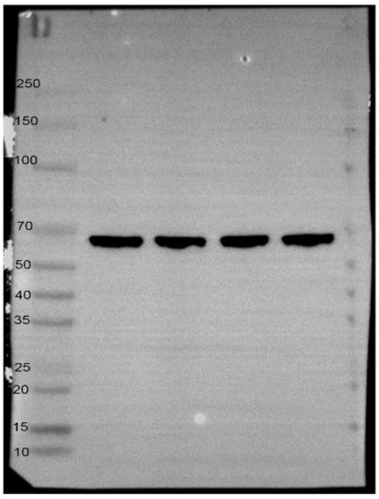

the second AKT

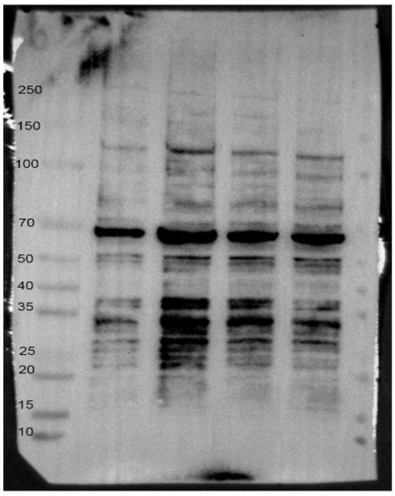

the second p-AKT

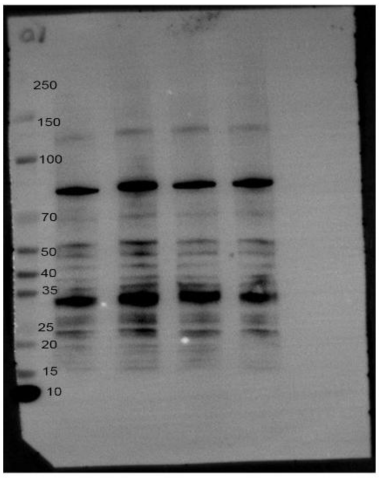

the second PI3K

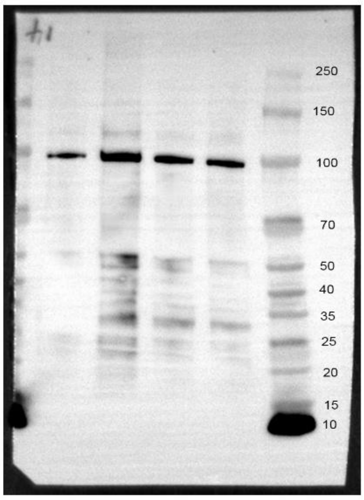

the second p-PI3K

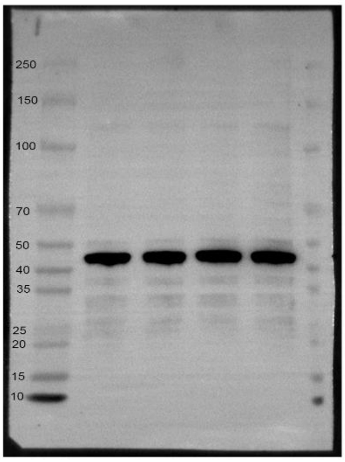

the second β-actin

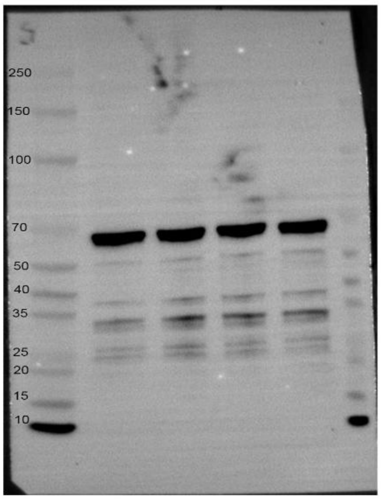

the third AKT

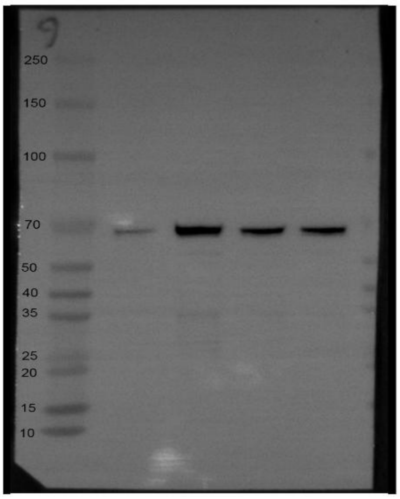

the third p-AKT

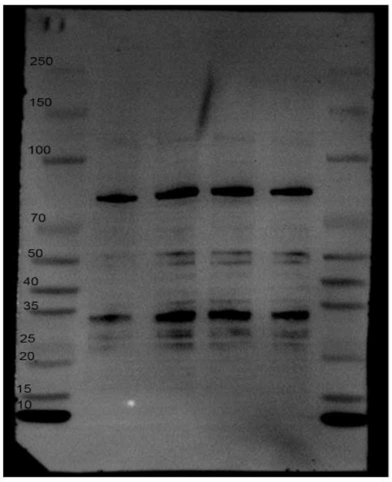

the third PI3K

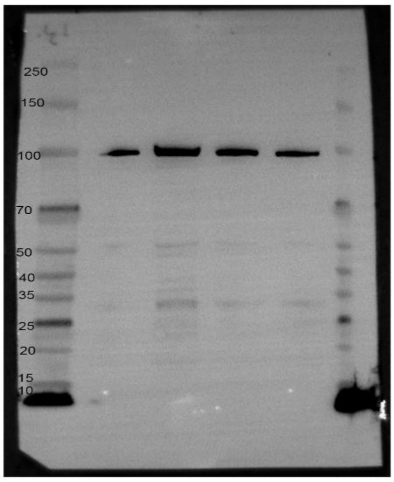

the third p-PI3K

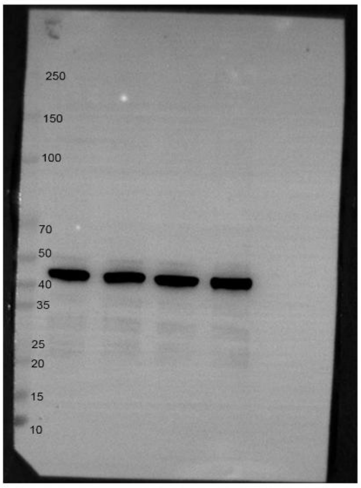

the third β-actin

Loading order: (left) control, model, TPL, LY294002
