# Supplementary figures and images for: Network pharmacology and experiments verify the effect of triptolide on extraocular muscle fibrosis
Source: PLoS One. 2025 Nov 10;20(11):e0336487. doi: 10.1371/journal.pone.0336487 (PMC12599947; doi:10.1371/journal.pone.0336487)

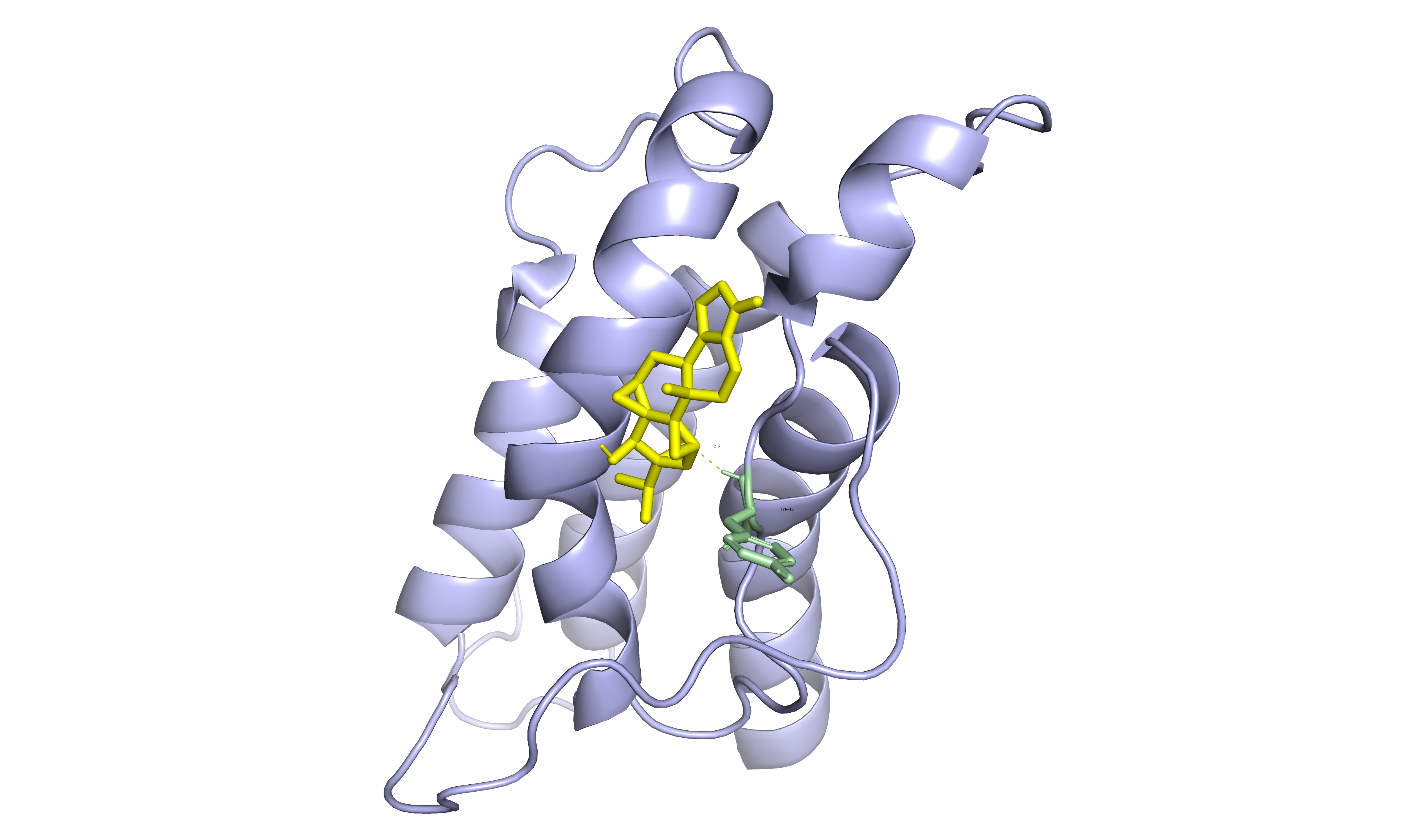

Supplement: S2 File — (ZIP) [file pone.0336487.s002.zip › S2_raw data/vina new/TPL dock/TPL IL2/2.png]

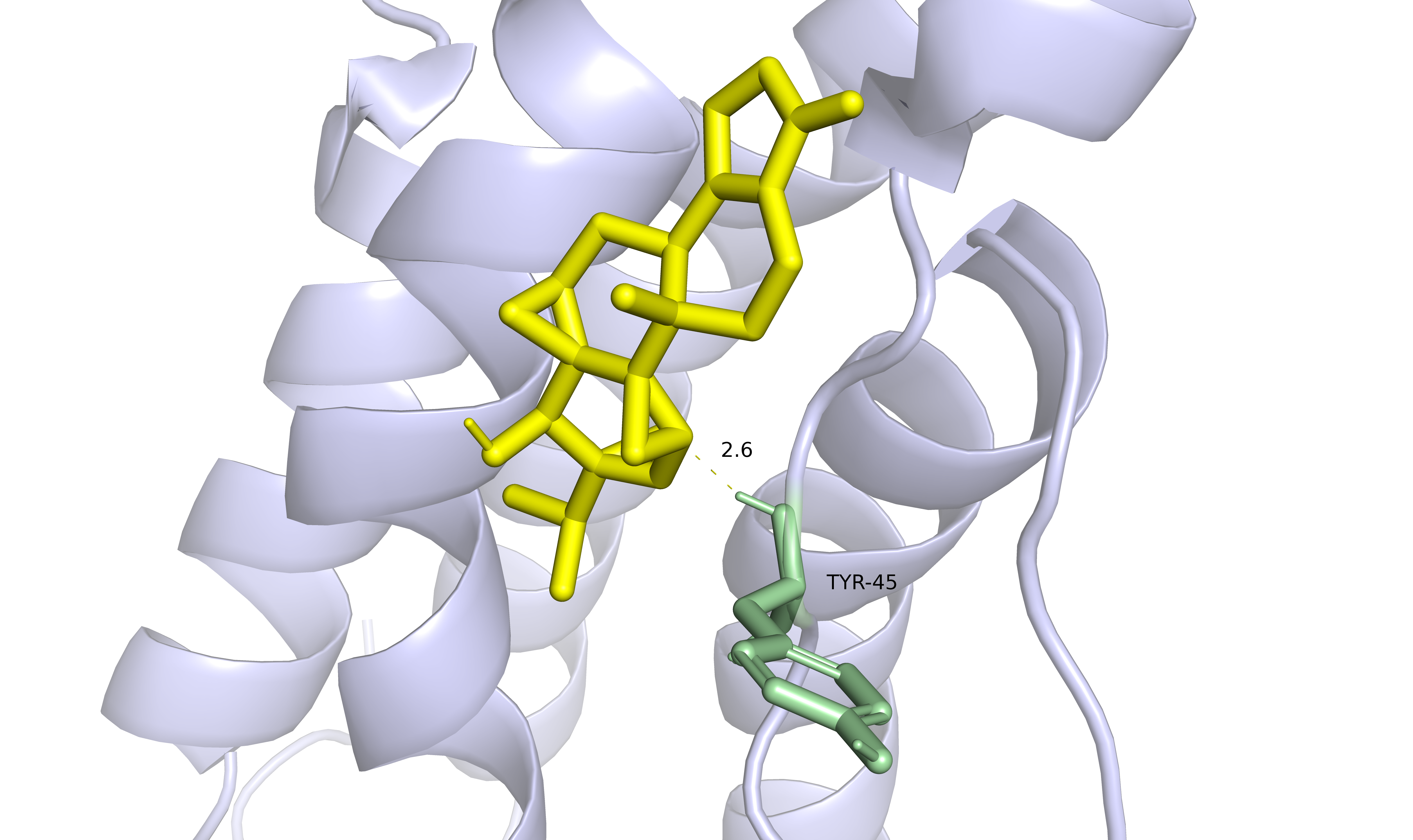

Supplement: S2 File — (ZIP) [file pone.0336487.s002.zip › S2_raw data/vina new/TPL dock/TPL IL2/3.png]

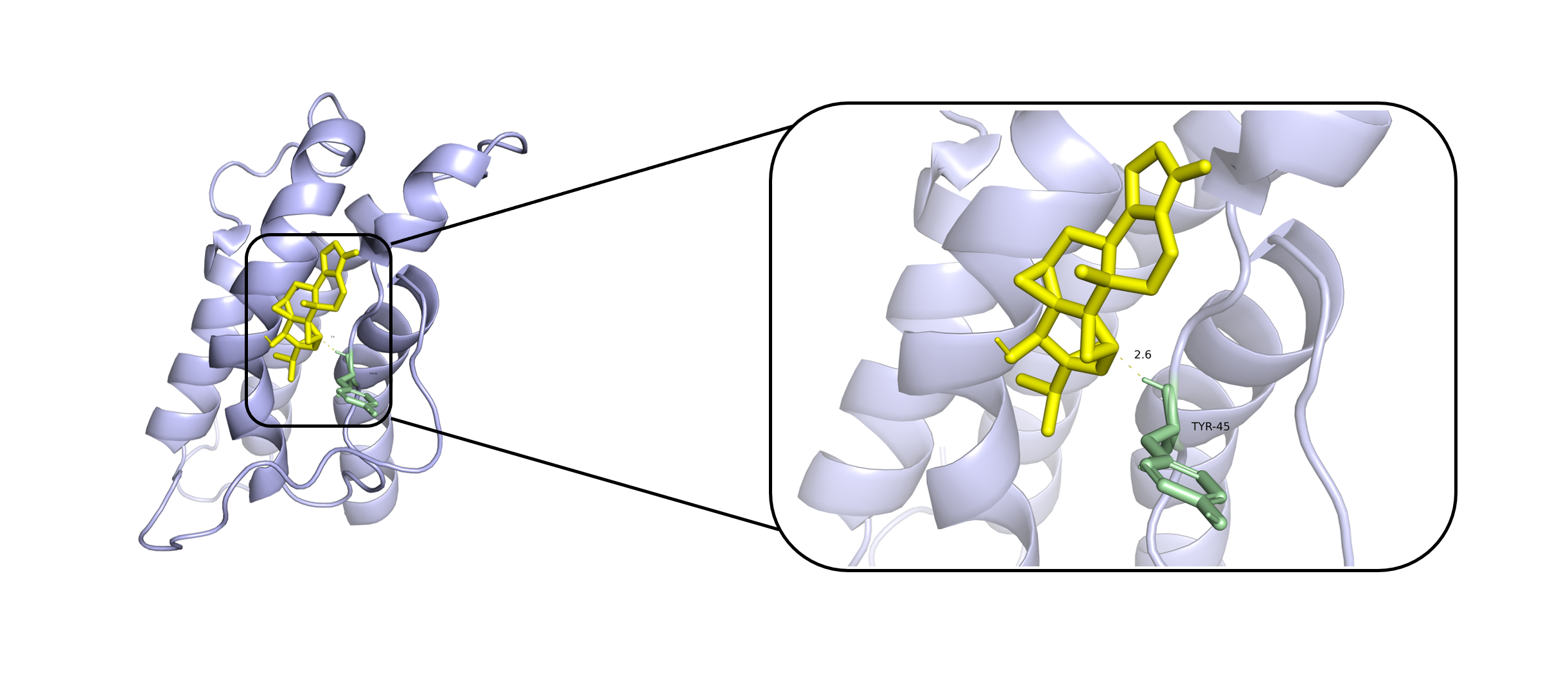

Supplement: S2 File — (ZIP) [file pone.0336487.s002.zip › S2_raw data/vina new/TPL dock/TPL IL2/Fig 8.tiff]

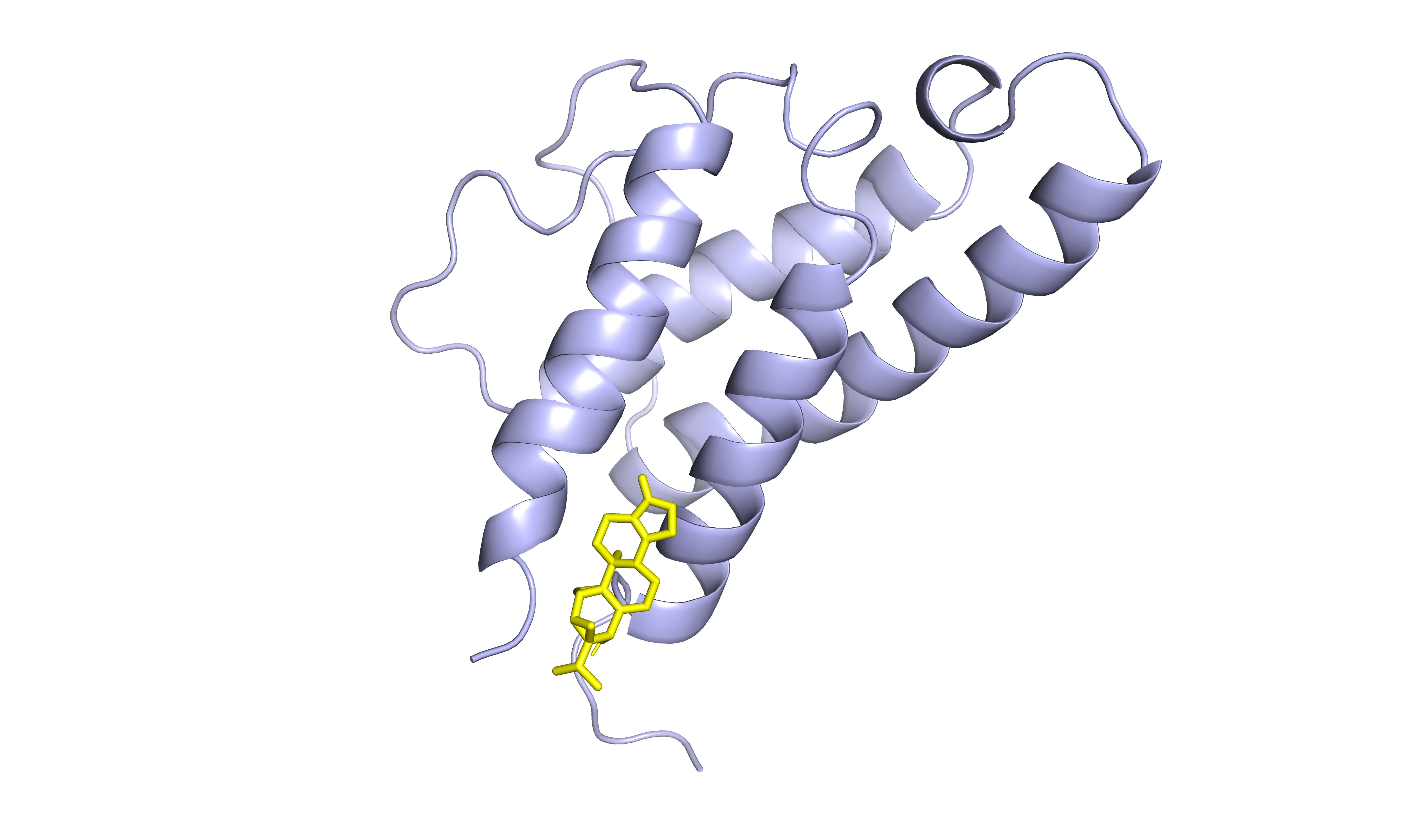

Supplement: S2 File — (ZIP) [file pone.0336487.s002.zip › S2_raw data/vina new/TPL dock/TPL IL4/2.png]

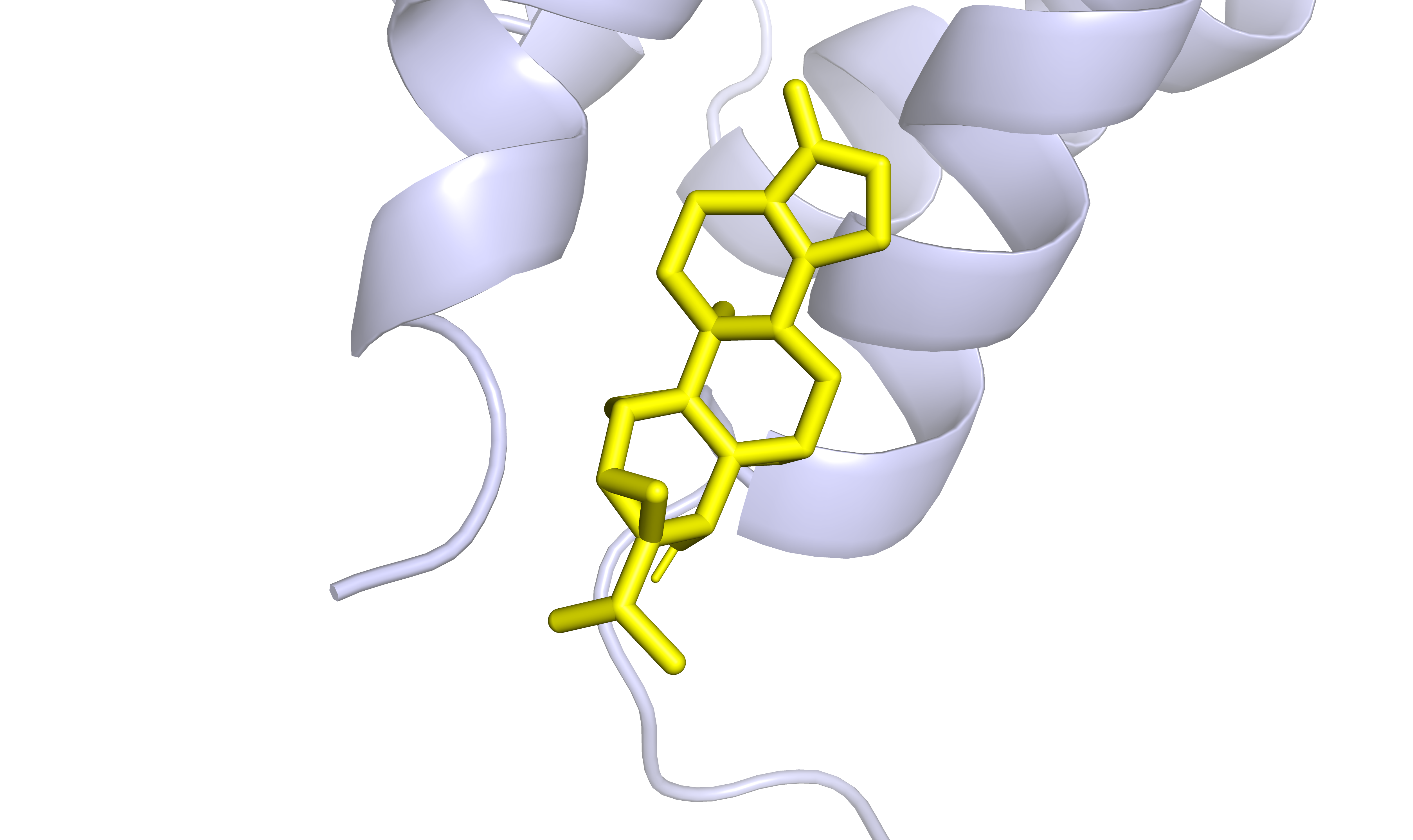

Supplement: S2 File — (ZIP) [file pone.0336487.s002.zip › S2_raw data/vina new/TPL dock/TPL IL4/3.png]

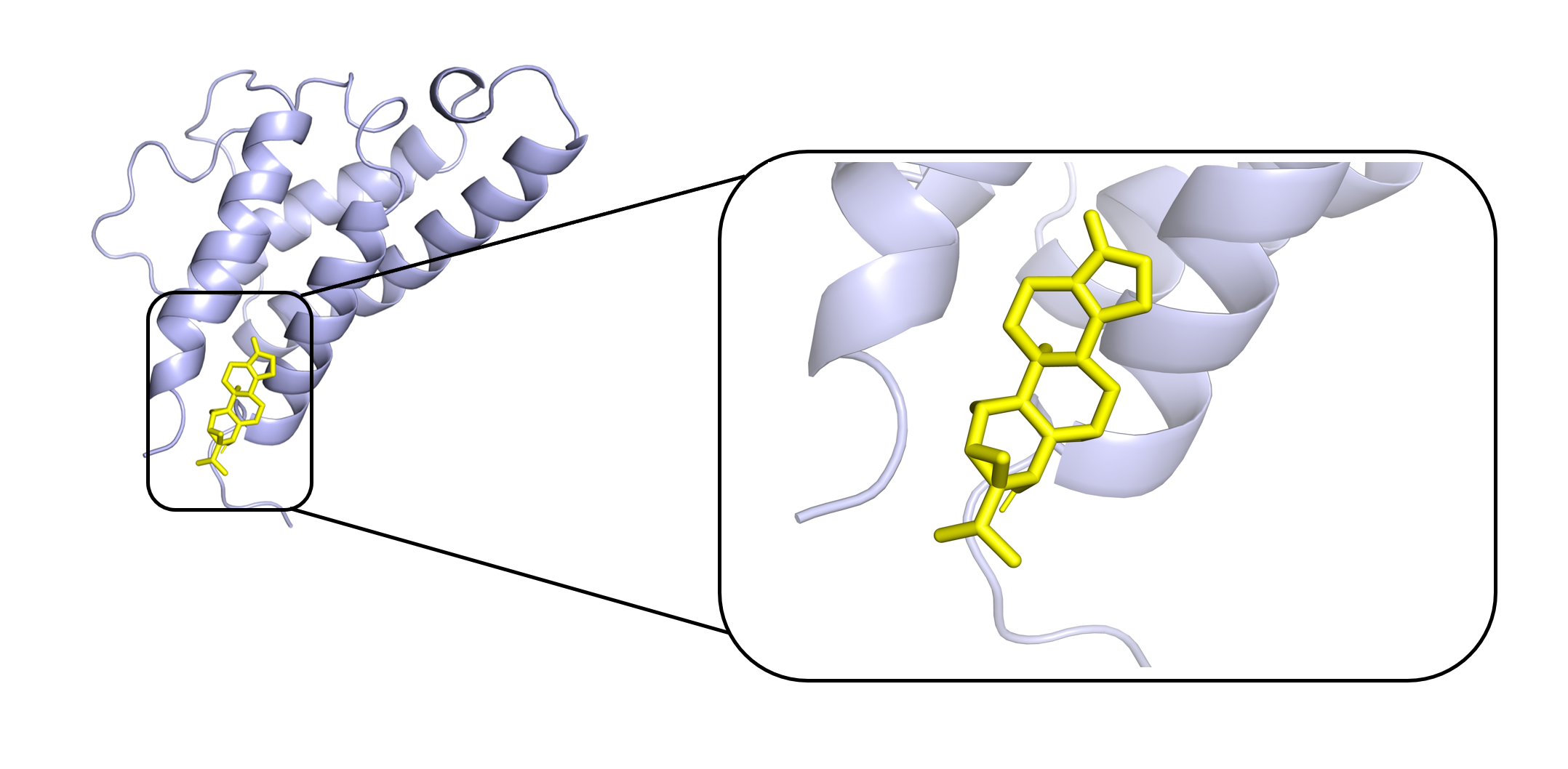

Supplement: S2 File — (ZIP) [file pone.0336487.s002.zip › S2_raw data/vina new/TPL dock/TPL IL4/Fig 9.tiff]

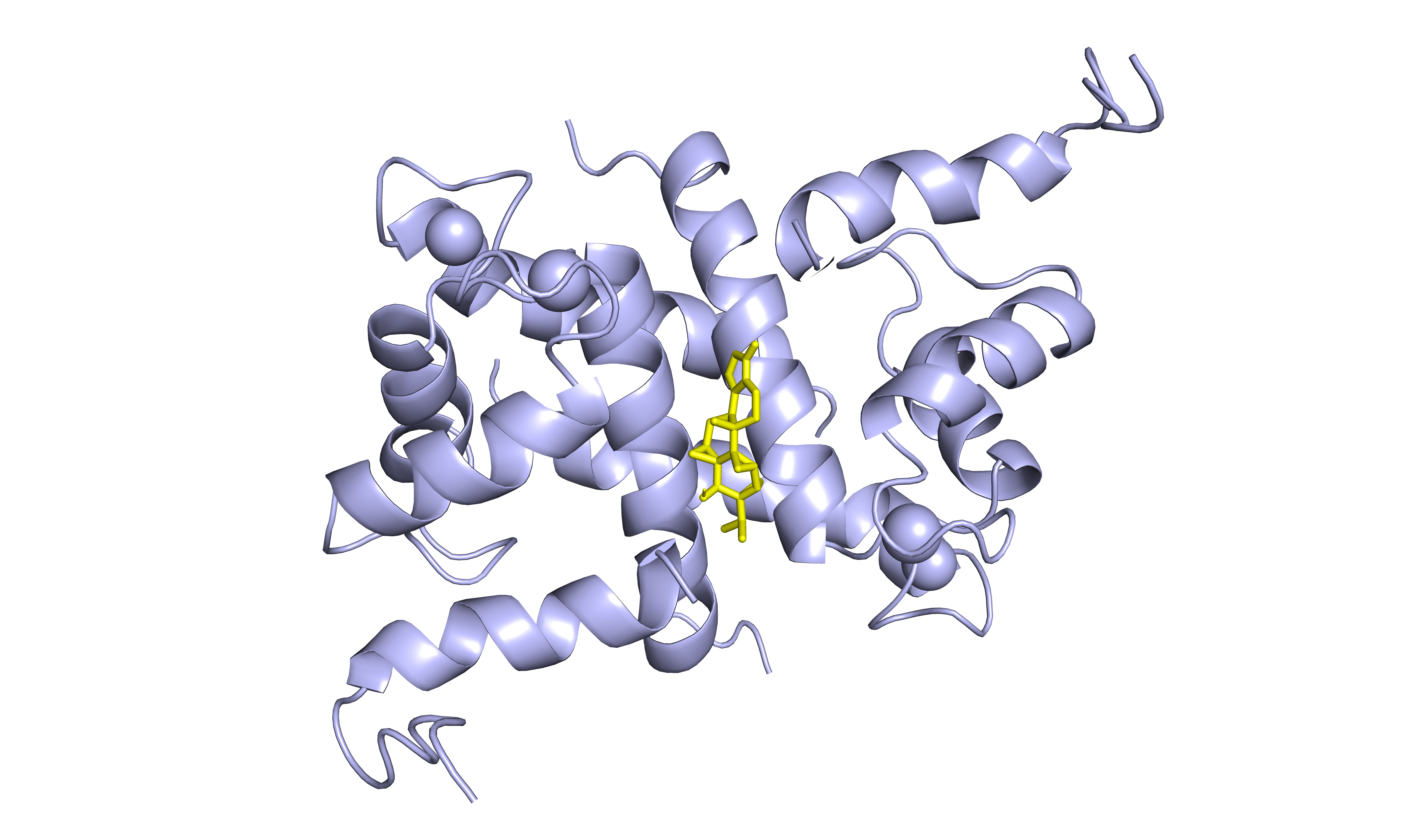

Supplement: S2 File — (ZIP) [file pone.0336487.s002.zip › S2_raw data/vina new/TPL dock/TPL TP53/2.png]

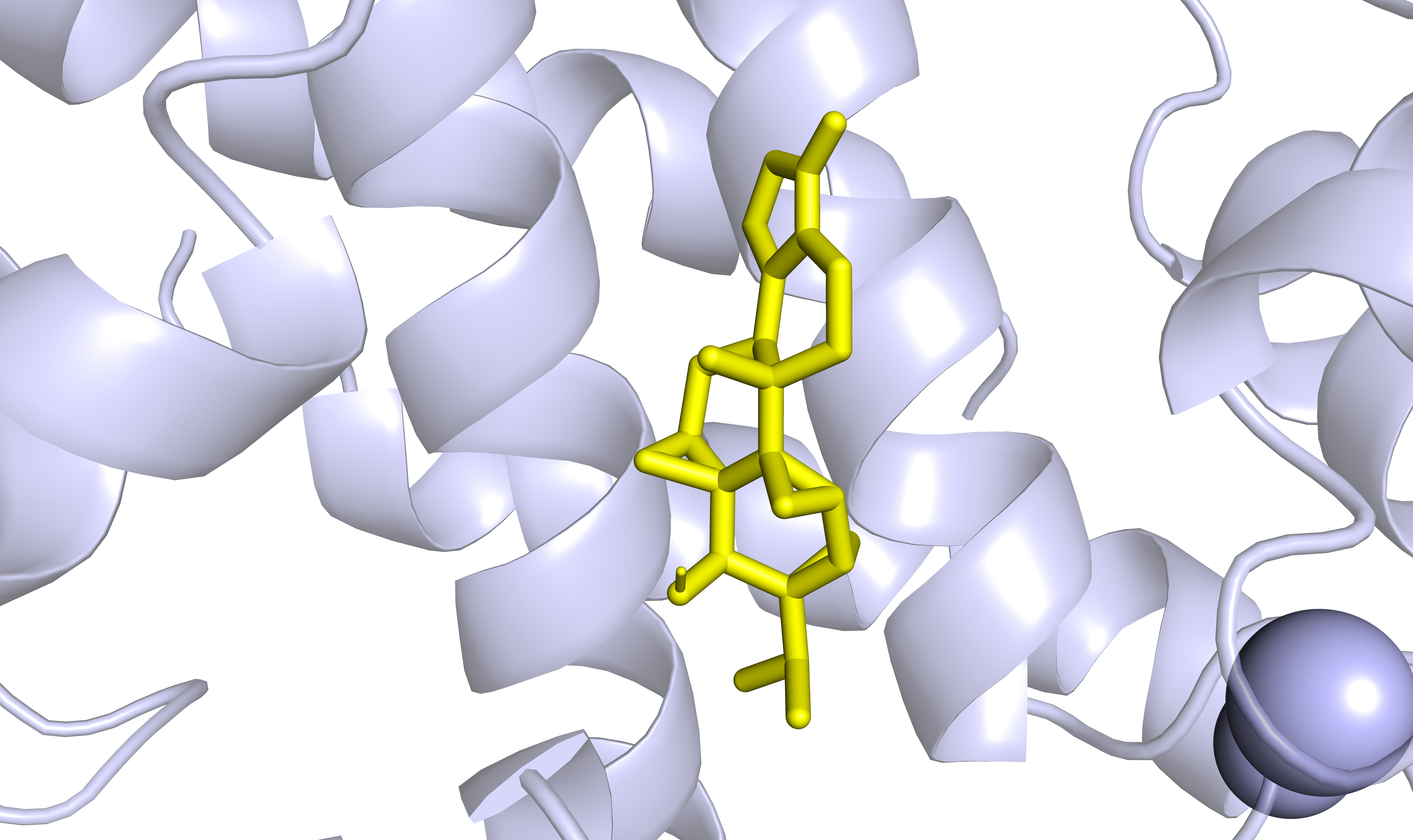

Supplement: S2 File — (ZIP) [file pone.0336487.s002.zip › S2_raw data/vina new/TPL dock/TPL TP53/3.png]

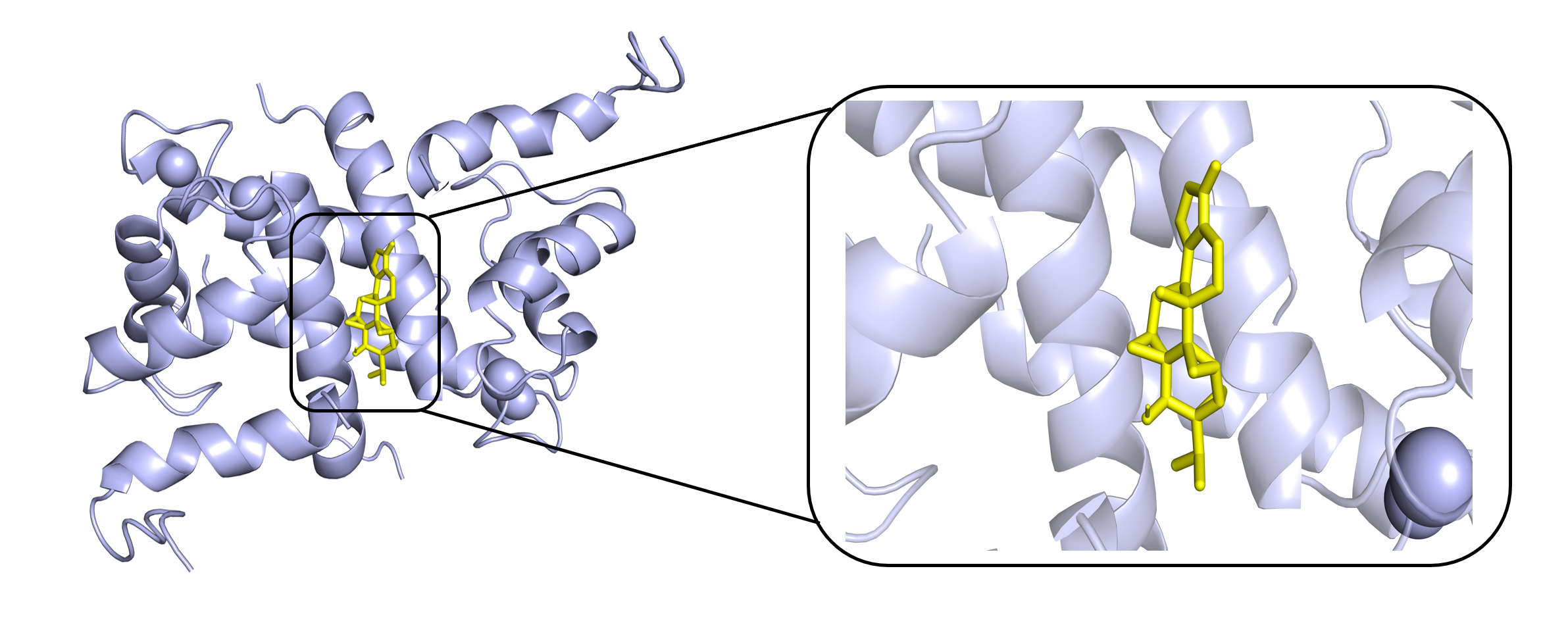

Supplement: S2 File — (ZIP) [file pone.0336487.s002.zip › S2_raw data/vina new/TPL dock/TPL TP53/Fig 7.tiff]

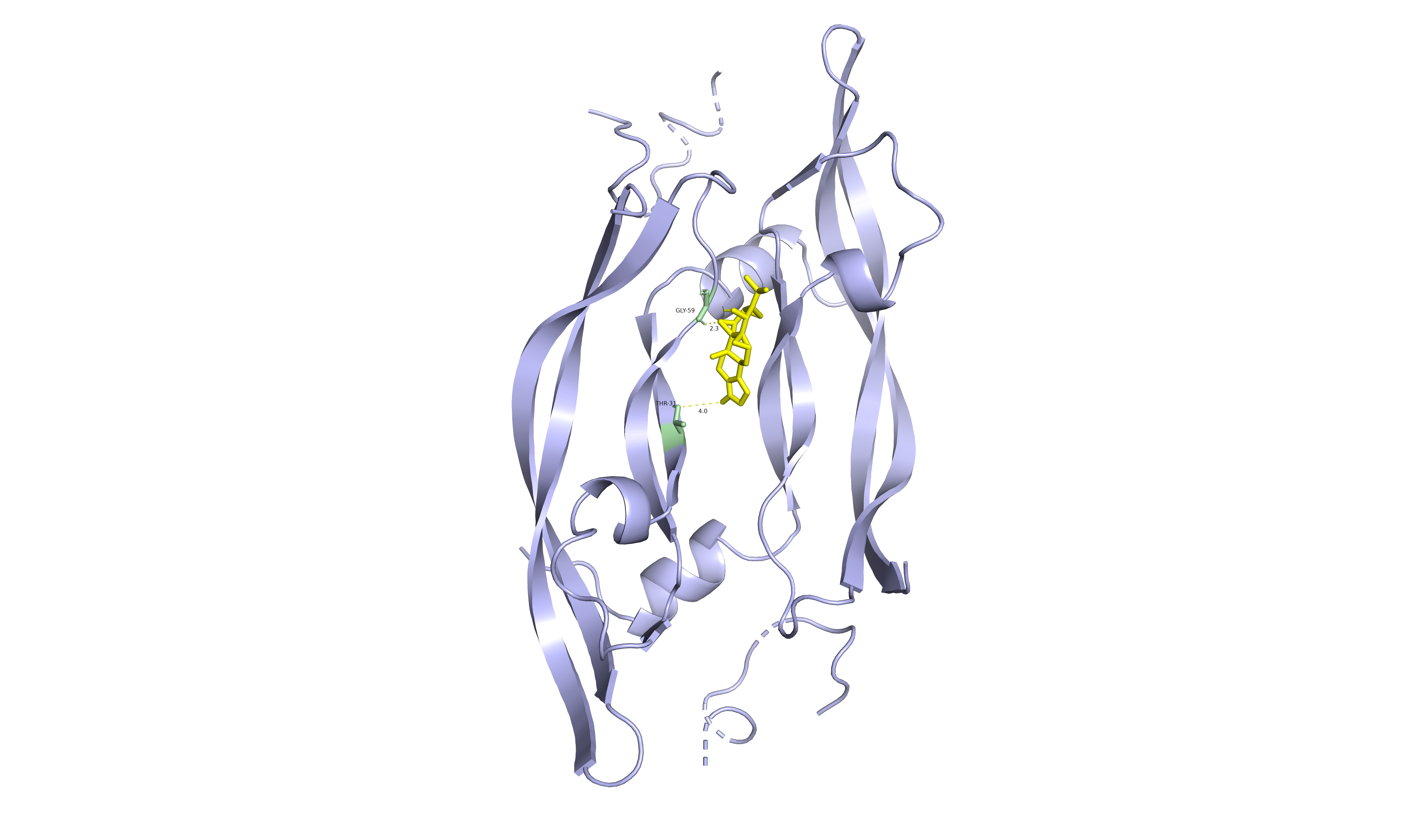

Supplement: S2 File — (ZIP) [file pone.0336487.s002.zip › S2_raw data/vina new/TPL dock/TPL VEGFA/2.png]

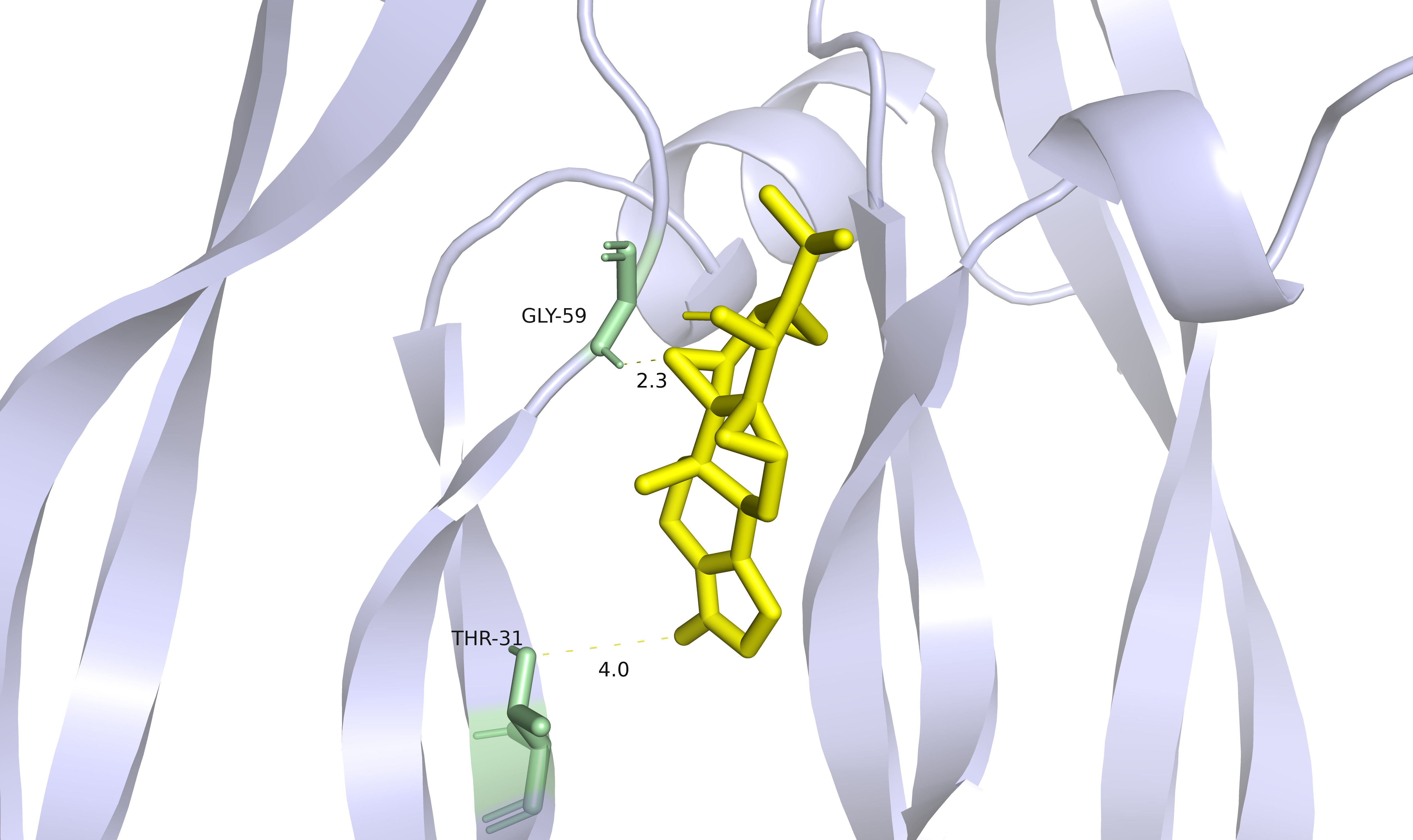

Supplement: S2 File — (ZIP) [file pone.0336487.s002.zip › S2_raw data/vina new/TPL dock/TPL VEGFA/3.png]

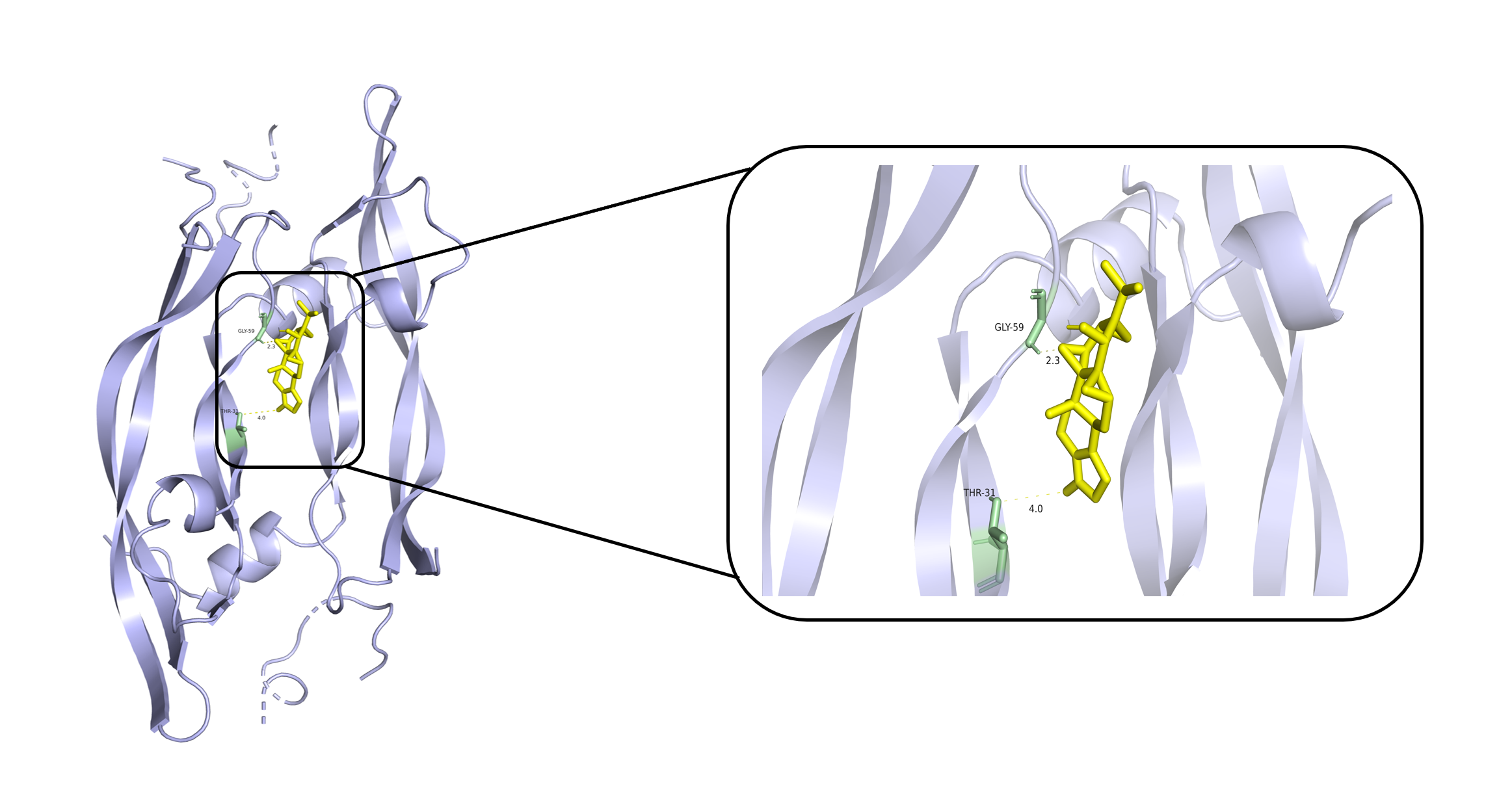

Supplement: S2 File — (ZIP) [file pone.0336487.s002.zip › S2_raw data/vina new/TPL dock/TPL VEGFA/Fig 10.tiff]
